# Aqueous Root Bark Extract of *Daniellia oliveri* (Hutch. & Dalz.) (Fabaceae) Protects Neurons against Diazepam-Induced Amnesia in Mice

**DOI:** 10.1155/2020/7815348

**Published:** 2020-07-21

**Authors:** Galba Jean Beppe, Lea Blondelle Kenko Djoumessie, Eglantine Keugong Wado, Hervé Hervé Ngatanko Abaïssou, Balbine Kamleu Nkwingwa, Jorelle Linda Damo Kamda, Roland Rebe Nhouma, Harquin Simplice Foyet

**Affiliations:** Department of Biological Sciences, Faculty of Science, University of Maroua, P.O. Box 814, Maroua, Cameroon

## Abstract

*Daniellia oliveri* (DO) is a traditional medicinal plant used for the treatment of diseases such as inflammation, schizophrenia, and epilepsy in Nigeria, Kenya, Congo, and Cameroon. This study was carried out to evaluate the potential neuroprotection effect of the aqueous root bark extract of *Daniellia oliveri* against diazepam-induced amnesia in mice. Thirty-six adult male mice were distributed into six groups: the three test groups received *Daniellia oliveri* root bark extract (100, 200, and 300 mg/kg), the normal control group received distilled water (10 ml/kg), a positive control group received piracetam (150 mg/kg), and the negative control received diazepam (2.5 mg/kg). Learning and memory were evaluated using the radial arm maze and the T-maze. Biomarkers of oxidative stress were also quantified in mice brains. Statistical analyses were performed using two-way ANOVA followed by Tukey's post hoc test. *Daniellia oliveri* root bark aqueous extract decreased the number of working memory errors and number of reference memory errors in amnesic mice evaluated in the radial arm maze. Also, an increase in glutathione activity and a decrease in malondialdehyde levels were noted in the hippocampi homogenate of the extract-treated mice as compared to the diazepam-demented but untreated group. Moreover, pretreatment with *Daniellia oliveri* aqueous root bark extract reversed the decrease in hippocampal cell density observed in the nontreated diazepam group. Taken together, these results suggest that the aqueous extract of DO leaves possesses antioxidant potential and might provide an opportunity for the management of neurological abnormalities in amnesic conditions.

## 1. Introduction

Amnesia is a neuropsychological pathology which results in the partial or total loss of memory. The pathology occurs when complex neurobiological processes, involved in learning and short- or long-term storage of information, are disrupted [1]. It is the main cause of dementia syndrome, which accounts for at least 70% of the cases [2]. The distribution of the new cases has reached 4.9 million (49%) in Asia, 2.5 million (25%) in Europe, 1.7 million (18%) in America, and 0.8 million (8%) in Africa [2]. Cameroon is not immune to this pathology with a prevalence of 2.85% in the city of Yaoundé [3]. Causes of this pathology are poorly known until now; however, age, environmental, and genetic factors are considered as risk factors to the occurrence of this disease.

Diazepam (DZP) is a benzodiazepine (allosteric modulators GABA receptor) which is well tolerated in organism and employed in the treatment of memory disorders, psychomotor disorders, sedation, dependence, and anxiety rebound [4]. Acute administration of DZP causes anterograde amnesia [5], while intravenous administration significantly impairs free recall based on the dose used [6]. Pretreatment with diazepam (2.0–16.0 mg/kg *i.p*.) or scopolamine (3.0 mg/kg *i.p*.) causes an alteration of the anterograde memory, in a dose-dependent manner [4, 7]. According to Maria et al. [8], intraperitoneal injection (*i.p*.) of 2.5 mg/kg DZP induces amnesia in rodents by hyperpolarization of neurons, decreased excitability, and increased lipid peroxidation [9, 10]. The current treatment options, such as surgery and synthetic drugs, are limited in their ability to improve neuronal function because they fail to repair damaged neurons or improve neural regeneration [11]. Due to the adverse side effects of standard antiamnesic drugs line, rivastigmine, donepezil, and galantamine, alternative therapies consisting of plant-derived medications, are increasingly being used to relieve neurodegenerative disorders [12].

*Daniellia oliveri* is a plant which grows in the Amazonia, certain part of America, and some Africans countries including Cameroon. Roots of this plant are used in traditional medicine to treat anxiety and schizophrenia [13]. Leaves decoction and stem bark infusion have been used as diuretic and aphrodisiac and applied as skin lotion. Dry leaves powder is also administered to treat yellow fever, back ache, and headache and for wound healing. The dried root and stem bark have been used in Ivory Coast as chewing stick and the water extract of the stem bark showed antibacterial activity [14]. Hexane and methanolic extracts of the bark showed analgesic and anti-inflammatory activities, while the methanolic extract of the stem bark showed smooth muscle relaxant activity [15]. Gaston and Daget [16] reported the presence of glycosides, flavonoids, saponins, rutin, quercitrin, and quercimeritrin in leaves. Saponins and flavonoids are recognized as anti-inflammatory and antioxidant compounds which could impart many health benefits and thereby reduce the risk of many chronic illnesses like degenerative disorder [17, 18]. This study was carried out to investigate the neuroprotective effects of *D. oliveri* root bark aqueous extract, in the diazepam-induced amnesia (DZP) mouse model.

## 2. Materials and Methods

### 2.1. Chemicals

Diazepam, acetylthiocholine iodide, 5,5-dithiobis (2-nitro-benzoic acid) (DTNB), 2-thiobabituric acid (TBA), and piracetam were purchased from Sigma-Aldrich, USA. All drugs and extracts were freshly prepared in saline on the day of experiments.

### 2.2. Plant Material and Plant Extract

*Daniellia oliveri* root bark was collected in Lara (Far North Region, Cameroon) in the month of December 2017 and authentificated at the National Herbarium, Yaoundé, where a voucher specimen was deposited under the reference number 14890/SRF/Cam-YA. After collection, *Daniellia oliveri* (DO) root bark was shade-dried for 5 days and pulverized into a fine powder. The extraction procedure used was as previously described by Soro et al. [19]. Briefly, 150 g pulverized sample material was introduced in 1 L of distilled water and boiled for 30 min. The filtrate was passed through a Whatman filter paper N° 4 and the solvent was eliminated from the extract using a temperature controlled oven (50°C for 24 hours). The powder obtained was weighed and the extraction yield was determined (1.85%).

### 2.3. Experimental Animals

Adult male Swiss mice aged 3 months (weighing 18–26 g) were obtained from the National Veterinary Laboratory of Garoua, Cameroon. Mice were acclimatized for 14 days, housed in polyacrylic cages (6 animals/cage) under natural dark-light cycle and provided with water and food *ad libitum*. Prior to and after treatment, mice were fasted for 12 and 7 h. Animal treatment and care was in accordance with the guidelines of the Cameroonian Bioethics Committee (Reg N° FWA-IRB00001954) and following NIH-Care and Use of Laboratory Animals Manual (8^th^ Edition). Each animal was tested in only one behavioral test and tests were made to minimize animal suffering.

**Figure 1:**
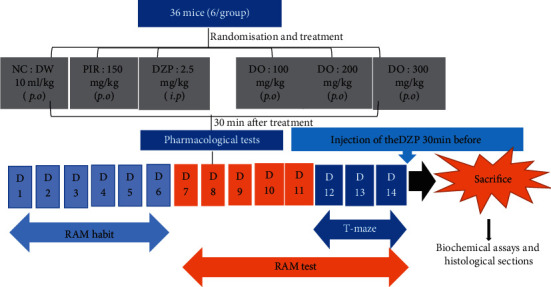
Experimental design. NC = neutral control, DW = distilled water, D = days, DO = *Daniellia oliveri*, and RAM = radial arm maze.

Mice were randomly divided into 6 groups of 6 animals each and subjected to the following treatment schedule (Figure 1). The normal control group received distilled water (DW) only (10 mL/kg *p.o*.), the negative control group received a single administration of diazepam (2.5 mg/kg, *i.p*. + DW) on day 14, the positive control group received a single dose of piracetam (150 mg/kg, *p.o*.), and different test groups received extract (DZP + 100, 200, and 300 mg/kg *p.o*. of DO) for 14 days. Diazepam was administered to all groups except the normal control 30 minutes after treatment on day 14. Behavioral tests were launched 30 minutes after diazepam administration.

### 2.4. Behavioral Test

#### 2.4.1. Radial Arm Maze Task

The radial arm maze used in the present study consisted of eight arms, numbered from 1 to 8 (48 cm × 12 cm), extending radially from a central area (32 cm in diameter). The apparatus was placed 50 cm above the floor. At the end of each arm, there was a food cup that had a single 50 mg food pellet. Before the performance of the maze task, animals were kept on restricted diet designed to keep their body weight at 85% of their free-feeding weight over a week, with water being available *ad libitum*. Before the actual training began, each mouse was simultaneously placed in the radial maze and allowed to explore for 5 min and take food freely. The food was initially available throughout the maze but was gradually restricted to the food cup. The animals were trained for 7 days to run to the end of the arms and consume the bait. The animals were trained for maze task performance by conducting daily training trials for 5 min, during which they do not receive any drug. The training trial continued until all the 4 baits had been consumed or until 5 min had elapsed. Completely trained animals were chosen for the study. Briefly, each animal was placed individually in the center of the maze and subjected to working and reference memory tasks, in which the same four arms (1, 3, 5, and 7) were baited for each daily training trial. The other four arms (2, 4, 6, and 8) were never baited. An arm entry was counted when all four limbs of the mouse were within an arm. Measures were made on the number of working memory errors (entering an arm containing food, but previously entered) and reference memory errors (entering an arm that was not baited). The time taken to consume all four baits was also recorded. Reference memory was regarded as a long-term memory for information that remains constant over repeated trials (memory for the positions of baited arms), whereas working memory was considered a short-term memory in which the information to be remembered changes in every trial (memory for the positions of arms that had already been visited in each trial) [20].

#### 2.4.2. T-Maze Task

The T-maze test evaluates the ability of the rodent to memorize its first choice. It is a behavioral approach to study the following aspects of cognition: alternation behavior, location recognition, object recognition, spatial discrimination, working memory, and reference memory which is measured here [21]. The T-maze used in this study consisted of a T-shaped device, made of wood, painted white. It consists of a departure compartment and two arrival arms 30 cm long, 10 cm wide, and 25 cm high. Aluminum doors were placed at the exit of the departure compartment and at the entrance of each arrival corridor to control access to the various areas of the maze. At the end of each arrival compartment was a small cup containing a bait.

Two days before the start of the experiments, animals were progressively deprived of food to maintain them at 85% of their weight. Mice were placed one after the other in the starting arm of the T-maze. After each trial, the maze was cleaned with 70% ethanol, to eliminate residual odours left by the preceding mice. This test was carried out over three days during which habituation, acquisition, and retention phases were assessed each day, respectively [21].

The habituation phase consisted of a single session of free-choice trial. A mouse was placed in the start arm and allowed to explore the maze for 5 min. The preferred and discriminated arms of each mouse were noted. During the acquisition phase which was performed the next day, mice were subjected to a forced-choice trial. The discriminated arm was blocked by a guillotine door. Once the animal was released from the starting arm, it was allowed to explore the maze by entering the preferred arm and returning to the start arm. During retention, all the guillotine doors were opened and mice were free to explore all the arms. In all the sessions, each mouse was evaluated for 5 min and the time spent in arms and number of returns to start arm were recorded. The floor of the apparatus was cleaned with 70% ethanol between trials to eliminate olfactory cues [22].

### 2.5. Biochemical Assay

After the behavioral tests, all mice were deeply anesthetized (using sodium pentobarbital, 100 mg/kg b.w., *i.p*.) and decapitated and whole brains were removed. The hippocampus was carefully excised. The hippocampal sample was homogenized (1 : 10) in ice-cold 0.1 M phosphate buffer (pH 7.4). The homogenate was centrifuged (15 min) and the supernatant was used to evaluate malondialdehyde (MDA) and reduced glutathione (GSH) levels.

#### 2.5.1. Determination of MDA

The level of lipid peroxides was measured by thiobarbituric acid reaction previously described by Ohkawa et al. [23]. 200 *μ*L of supernatant added and briefly mixed with 1 mL of 50% trichloroacetic acid in 0.1 M HCl and 1 mL of 26 mM thiobarbituric acid after the vortex mixing sample was maintained at 95°C for 20 min. Furthermore, samples were centrifuged at 9609 rpm for 10 min and supernatants were read at 532 nm. A calibration curve was constructed using MDA as standard and the results were expressed as nmol/organ of tissues.

#### 2.5.2. Determination of GSH

It was measured following the method described by Fukuzawa and Tokumura [24]. 200 *μ*L of supernatant was added to 1.1 mL of 0.25 M sodium phosphate buffer (pH = 7.4) followed by the addition of 130 *μ*l DTNB 0.04%. Finally, the mixture was brought to a final volume of 1.5 mL with distilled water and absorbance was read in a spectrophotometer at 412 nm and results were expressed as *μ*g GSH/*μ*g organ.

### 2.6. Histology of the Tissues

On day 14, after the acquisition of hippocampi, whole brains were collected, fixed in 10% formalin for a week. Fifty (50) mm coronal sections were made from the brain in the hippocampus region using the Mouse Brain Atlas with the following coordinate (anterior/posterior D 2.0 mm, medial/lateral D 1.5 mm, and dorsal/ventral AP D 2.0 mm) [25]. Hippocampi sections were collected in nine-well plates. The dehydration of these sections consisted in introducing tissues in ascending concentrations of ethanol followed by immersion in xylol and then embedding in paraffin. Paraffin sections of the brain were deparaffinised and rehydrated through washes in descending concentration series of alcohol. Hippocampi were then stained using hematoxylin and eosin stains. The brain sections were thereafter photographed and images were captured using a digital camera attached to a light microscope (Scientico, Haryana, India).

### 2.7. Statistical Analysis

All the results were expressed as mean ± SEM. The data were analyzed by one-way ANOVA (T-maze) and two-way ANOVA (RAM) followed by Tukey and Bonferroni post hoc tests, respectively. All analyses were performed using Graph Pad Prism 5.00 software, San Diego, California, USA. Results were considered significant at *p* < 0.05.

## 3. Results

### 3.1. Effect of *D. oliveri* Root Bark Aqueous Extract on Working and Spatial Reference Memory in the Radial Arm Maze

**Figure 2:**
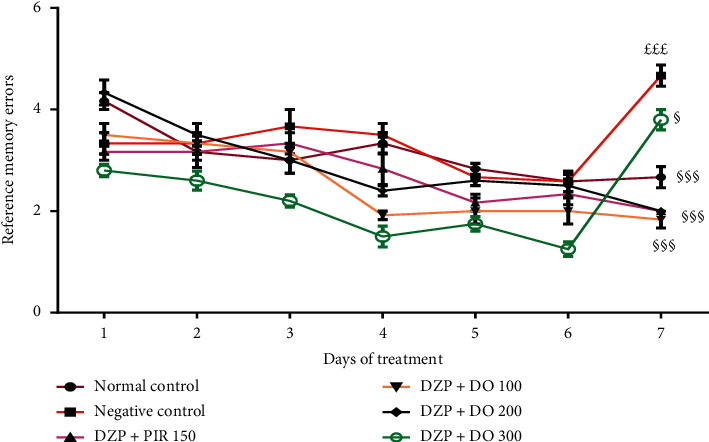
Effect of the aqueous extract of *D. oliveri* root on the reference memory of amnesic mice in the radial maze test. Each bar represents the mean ± ESM, *n* = 6. ^£££^*p* < 0.001 = significant difference compared to the normal control; ^§^*p* < 0.05 and ^§§§^*p* < 0.001 = significant difference compared to negative control. DZP 2.5 + DW = diazepam (2.5 mg/kg) + distilled water; PIR 150 = piracetam (150 mg/kg); DO 100, 200, 300 = *Daniellia oliveri* (100, 200, 300 mg/kg).

**Figure 3:**
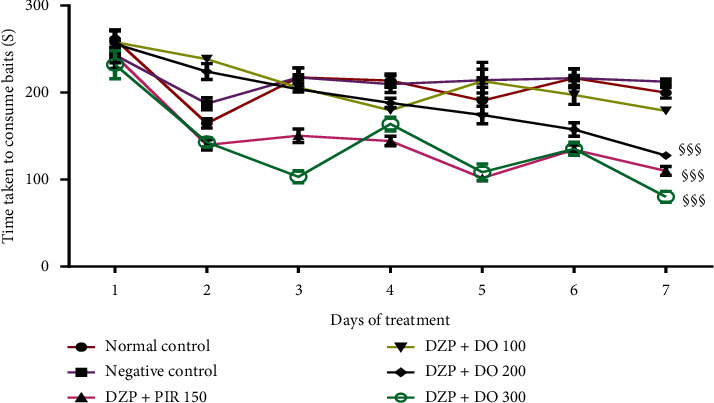
Effects of the aqueous root extract of *D. oliveri* on the time taken to consume the baits in the radial maze test. Each bar represents the mean ± ESM, *n* = 6. ^§§§^*p* < 0.001 = significant difference compared to the negative control. DZP 2.5 + DW = diazepam (2.5 mg/kg) + distilled water; PIR 150 = piracetam (150 mg/kg); DO 100, 200, and 300 = *Daniellia oliveri* (100, 200, 300 mg/kg).

To investigate whether the aqueous extract of *D. oliveri* (100, 200, and 300 mg/kg) affects spatial memory formation, the mice were evaluated in the radial arm maze task. For reference memory errors, ANOVA revealed a significant time difference (*p* < 0.01) (Figure 2). Additionally, Tukey's post hoc analysis revealed significant differences between the normal control and DZP groups (*p* < 0.01), normal control and DZP + DO (100 mg/kg) groups (*p* < 0.01), DZP and DZP + DO (200 mg/kg) groups (*p* < 0.01), and DZP and DZP + DO (300 mg/kg) groups (*p* < 0.01). There were no significant differences between different doses of extract (*p* > 0.05). Moreover, the time taken to consume all the baits significantly increased (*p* < 0.001) in untreated mice compared to the normal control group (Figure 3). The administration of *D. oliveri* root back aqueous extract significantly (*p* < 0.001) reduced this time in DZP + DO (100 mg/kg) groups (*p* < 0.001), DZP + DO (200 mg/kg) groups (*p* < 0.01), and DZP + DO (300 mg/kg) groups (*p* < 0.001) compared to DZP group.

**Figure 4:**
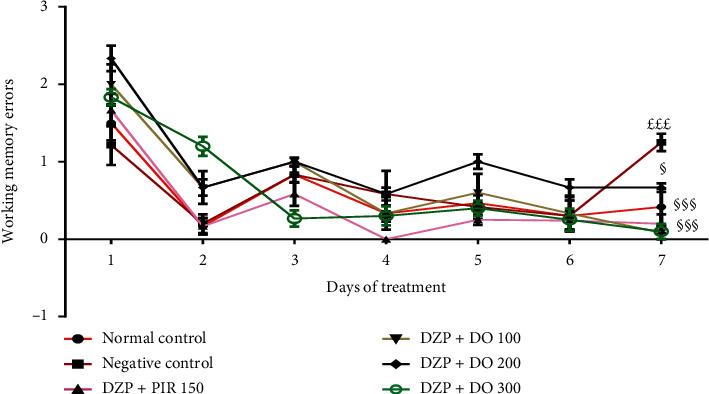
Effects of the aqueous extract of *D. oliveri* root bark on the number of working memory errors on in the radial maze test. Each bar represents the mean ± ESM, *n* = 6. ^£££^*p* < 0.001 = significant difference compared to the normal control; ^§§§^*p* < 0.001 = significant difference compared to the negative control. DZP 2.5 + DW = diazepam (2.5 mg/kg) + distilled water; PIR 150 = piracetam (150 mg/kg); DO 100, 200, and 300 = *Daniellia oliveri* (100, 200, and 300 mg/kg).

For working memory errors, Tukey's post hoc analysis revealed significant differences between the normal control and DZP groups (*p* < 0.001), DZP and DZP + DO (100 mg/kg) groups (*p* < 0.001), DZP and DZP + DO (200 mg/kg) groups (*p* < 0.001), and DZP and DZP + DO (300 mg/kg) groups (*p* < 0.001) on the 7^th^ day of the treatment (Figure 4).

### 3.2. Effect of *D. oliveri* Root Bark Aqueous Extract on Long-Term Memory in T-Maze Task

**Figure 5:**
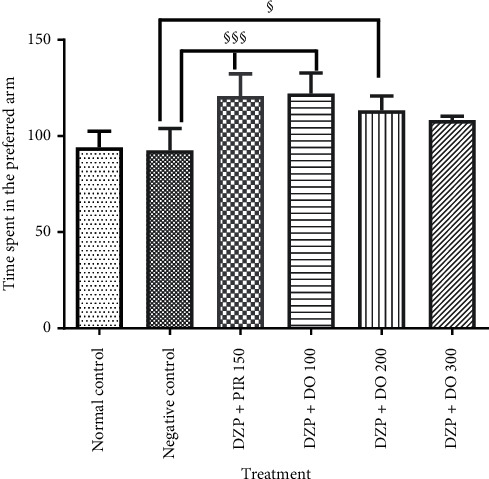
Effects of the aqueous extract of *D. oliveri* roots on the time spent in the preferred arm of the T-maze test. Each bar represents the average ± ESM, *n* = 6. ^§^*p* < 0.05 and ^§§§^*p* < 0.001 = significant difference compared to negative control. DZP 2.5 + DW = diazepam (2.5 mg/kg) + distilled water; PIR 150 = piracetam (150 mg/kg); DO 100, 200, and 300 = *Daniellia oliveri* (100, 200, and 300 mg/kg).

**Figure 6:**
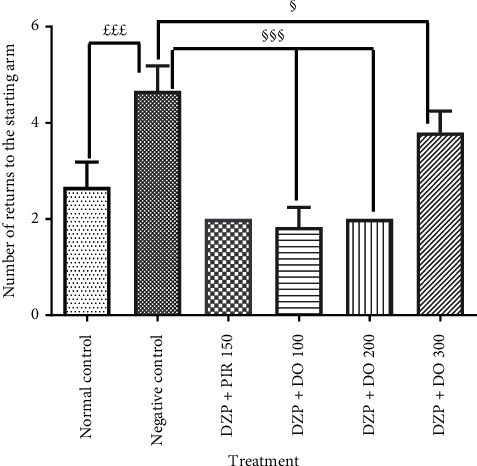
Effects of the aqueous extract of *D. oliveri* roots on the number of starting arm return of the T-maze test. Each bar represents the mean ± ESM, *n* = 6. ^£££^*p* < 0.001 significant difference compared to the normal control; ^§§§^*p* < 0.001 and ^§^*p* < 0.05 significant difference compared to negative control. N control = normal control; DZP 2.5 + DW = diazepam (2.5 mg/kg) + distilled water; PIR 150 = piracetam (150 mg/kg); DO 100, 200, and 300 = *Daniellia oliveri* (100, 200, and 300 mg/kg).

The T-maze test was performed to confirm the potential effect of DO on long-term memory. ANOVA revealed that acute administration of diazepam significantly (*p* < 0.05) increased the time spent in the preferred arm as compared to the control group (Figure 5). Animals treated with *D. oliveri* aqueous extract 200 mg/kg for 14 days resulted in a significant (*p* < 0.05) increase of this time in the preferred arm compared to demented group. The treatment of animals with piracetam significantly increased (*p* < 0.001) the time spent in preferred arm in comparison with the untreated group. In addition, the number of returns in the starting arm increased significantly (*p* < 0.001) in untreated animals compared to the normal control ones (Figure 6). It was also observed that the administration of *D. oliveri* root bark aqueous extract, 100 and 200 mg/kg, significantly (*p* < 0.001) reduced these numbers as compared to the untreated group. No significant (*p* > 0.05) difference was noted with the 300 mg/kg doses of the extract.

### 3.3. Effect of DO on MDA Content and Reduced GSH Level

**Figure 7:**
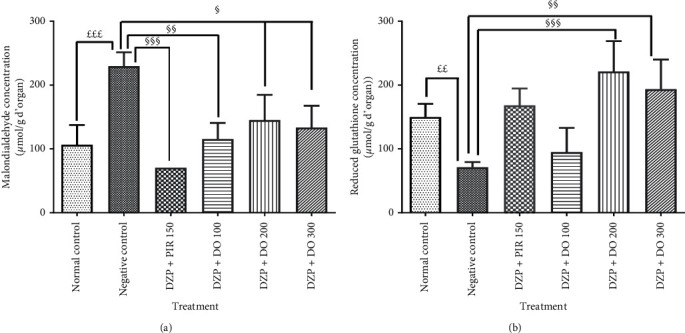
Effects of the aqueous extract of the root bark of *Daniellia oliveri* on the brain concentration of MDA (a) and reduced glutathione (b). Each bar represents the mean ± ESM, *n* = 6. ^£££^*p* < 0.001; significant difference compared to the normal control; ^§^*p* < 0.05, ^§§^*p* < 0.01, and ^§§§^*p* < 0.001; significant difference compared to the negative control. DZP 2.5 + DW = diazepam (2.5 mg/kg) + distilled water; PIR 150 = piracetam (150 mg/kg); DO 100, 200, and 300 = *Daniellia oliveri* (100, 200, and 300 mg/kg).

Brain malondialdehyde (MDA) concentration was significant (*p* < 0.001) in the negative control group mice compared to the normal control (Figure 7(a)). The treatment of the animals with the aqueous extract of *D. oliveri* at dose of 100 mg/kg for 14 days significantly (*p* < 0.01) reduced the concentration of MDA compared to animals receiving diazepam.

The hippocampal concentration of malondialdehyde was significantly (*p* < 0.001) decreased in the animals treated with piracetam in comparison with the untreated group.

Glutathione concentration (GHS) decreases nonsignificantly (*p* > 0.05) in the group of mice that received diazepam with respect to normal control mice (Figure 7(b)). The pretreatment of mice with *Daniellia oliveri* root bark aqueous extract 200 mg/kg for 14 days significantly (*p* < 0.001) increased this concentration in comparison with the demented group. This parameter was also increased nonsignificantly (*p* > 0.05) in animals that were given piracetam compared to the demented group which was not subjected to any treatment.

### 3.4. Effects of *Daniellia oliveri* on Hippocampi Histological Sections

**Figure 8:**
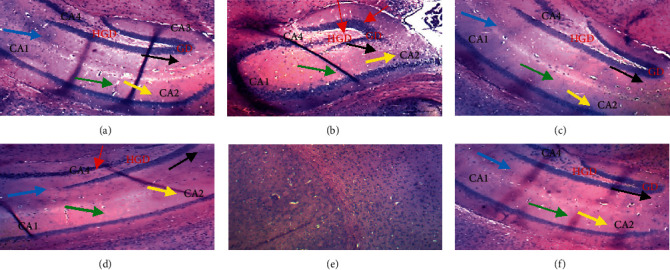
Effects of *D. oliveri* extract on hippocampi sections of mice brains. Coronal sections: hematoxylin and eosin-40x magnification; CA = cornus ammonis, GD = dentate gyrus, and HGD: dentate gyrus hilus. Yellow arrow: stratum oriens; green arrow: layer of pyramidal cells (contains the cell bodies of the pyramidal cells of the cornus ammonis. The density of the blue color corresponds to the density of the cell bodies of pyramidal neurons); blue arrow: stratum lacunosum-moleculare (contains pyramidal cell segments); black arrow: molecular layer of the dentate gyrus. ML: molecular layer of dentate gyrus. (a) Normal (DW 10 ml/kg). (b) DZP 2.5 mg/kg + DW. (c) DZP + PIR 150 mg/kg. (d) DZP + DO 100 mg/kg. (e) DZP + DO 200 mg/kg. (f) DZP + DO 300 mg/kg.

The effects of *Daniellia oliveri* aqueous root bark extract administration on the micromorphology of the hippocampus are illustrated in Figure 8. The analysis of the histological sections performed shows that in animals treated with DZP and receiving distilled water, the layer of granular cells at the dentate gyrus exhibits an abnormal architecture with some signs of cellular degeneration (red arrow) compared to that of the healthy control. In animals treated with DZP but did not receive the plant extract at doses of 100 and 300 mg/kg, the coronal sections revealed a normal architecture. However, signs of cellular degeneration still persist in the coronal section of animals treated with plant extract at a dose of 100 mg/kg.

## 4. Discussion

The aim of this study was to evaluate the neuroprotective effects of the aqueous extract of *Daniellia. oliveri* root bark on diazepam-induced amnesia model in mice. To achieve this, behavioral tests such as the radial maze and the T-maze were used, oxidative stress parameters (malondialdehyde and reduced glutathione) were evaluated in hippocampi homogenates, and the histological sections of the hippocampus were performed.

Diazepam is an anxiolytic and hypnotic which binds to GABA-A receptors to exert its effects [26, 27]. However, it is possible to experimentally induce amnesia, which is either temporary (in the case of episodic memory) or reversible (for semantic memory) with benzodiazepines [28]. The results of this work show an increase of the number of reference memory errors (entering an arm that was not baited) and working memory (entering an arm containing food, but previously entered) in diazepam-treated animals compared to animals in the neutral control group in the radial maze. Also, an increase was noted in the number of returns to the starting arm and a decrease of the time spent in the preferred arm in the diazepam-treated animals compared to the neutral control animals when they were subjected to the T-maze test. An increment in the time spent in the discriminated arm and a decrement in the time spent in the preferred arm are indications of impaired memory [29]. These observations were completely reversed by pretreatment with the aqueous extract of *D. oliveri* for 14 days. In fact, in the radial maze test, the aqueous extract of *D. oliveri* roots bark at different tested doses significantly (*p* < 0.001) reduced the number of working and reference memory errors, thus translating an improvement of the working and reference memory, respectively, in amnesic mice. In addition, there was a decrease (*p* < 0.001) in the time taken to consume all baits. Moreover, in the T-maze, the results obtained show a significant increase (*p* < 0.05) in the time spent in the preferred arm and a significant decrease (*p* < 0.001) in the number of returns to the starting arm with all the doses of the extract. According to Chapouthier et al. [30], the increase in the time spent in the preferred arm and a reduction in the time spent in the discriminated arm reflect a good functioning of the memory. Furthermore, Farshchi et al. [29] reported that a decrease in the number of returns to the starting arm suggests an improvement in memory. These observations suggest that the aqueous extract of *D. oliveri* roots could play an important role in memorization and more specifically by improving short-term and long-term memory. Pervious study has reported the presence of saponins, flavonoids, tannins, and alkaloids in the extract of *D. Oliveri.* These compounds are endowed with numerous pharmacological properties [16]. Indeed, Diaby [31] demonstrated that the aqueous extract of *Daniellia oliveri* bark had antiepileptic properties. In addition, the oleoresin isolated from this plant is an anti-inflammatory and antioxidant agent [32]. Therefore, the neuroprotective effect of this extract could be attributed to these groups of compounds. In order to elucidate how the extract act, the aqueous extract of the root bark of *Daniellia oliveri*, biochemical assays were carried out in homogenates of the hippocampus of amnesic mice.

DZP induces amnesia by generating reactive oxygen species [7]. The brain has increased susceptibility to oxidative stress because it contains high concentrations of polyunsaturated fatty acids vulnerable to lipid peroxidation and also has modest antioxidant capacity [33]. In addition, the high oxygen consumption in the brain, the metabolism of catecholamines, and the release of neurotransmitters are considered as important sources of free radicals and are therefore associated with significant oxidative damage [4]. Free radicals and related molecules are often classified together as reactive oxygen species (ROS) [33]. The formation of ROS in nerve cells is numerous, so cells must maintain an effective antioxidant system to protect against the overload of ROS and the resulting damage [7]. During oxidative stress, the production of ROS increases and exceeds the capacity of endogenous free radical scavengers such as GSH, CAT, and SOD [9, 10]. It has also been reported that ROS formation is involved in the neurotoxicity of many xenobiotics [4]. In this research, diazepam caused a significant change in the MDA content and GSH activities in the hippocampus. This observation was completely reversed by the pretreatment of animals with DO extract. However, concomitant administration of *D. oliveri* and diazepam not only reduced MDA concentration but also increased GSH concentration and enzymatic activity in the hippocampus of mice. GSH, CAT, and SOD are major enzymes that fight free radicals in the body. Indeed, GSH is vital for detoxifying heavy metals; the thiol group reacts with the salts of these heavy metals, creating with them a very strong sulfur-metal bond so that they are subsequently excreted without causing any harm to the organism [34]. The observed effect may be due to the radical scavenging properties of flavonoids contained in *D. oliveri*. The present results thus demonstrated the correlation between the antiamnesic effects of the aqueous extract of *D. oliveri* root bark against diazepam-induced amnesia and its antioxidant capacity.

Neurons provide the transmission of a bioelectric signal called nerve impulse. They have two physiological properties: excitability, that is, the ability to respond to stimuli and convert them into nerve impulses and conductivity, that is to say, the ability to transmit impulses nervous [35]. Any disturbance (degeneration) of a neuron at the level of the hippocampus leads to difficulty or inability to learn [36]. The histological section results showed a decrease in the density of the neurons of animals in the group receiving only diazepam compared to animals in the neutral control group. In contrast, DO (300 mg/kg) treated animals' hippocampi were protected from cell death associated to diazepam administration. The aqueous extract of *D. oliveri* root barks at a dose of 300 mg/kg therefore protects the neurons against diazepam-induced amnesia in mice. The aqueous extract of *D. oliveri* root bark could therefore act either as a DZP receptor antagonist or by inhibiting lipid peroxidation while increasing the antioxidant status. All these properties would be attributable once again to the various compounds (glycosides, flavonoids, rutin, quercitrin, tannins, saponins, and glycosides) contained in this plant. Stud phytochemical screening is still going on to identify and specify the compounds responsible of the observed effects.

## 5. Conclusion

The present study strongly demonstrates that *D. oliveri* root bark aqueous extract of 200 and 300 mg/kg effectively enhances memory processes, restores antioxidant brain status, and may confer neuroprotection by the alleviation diazepam-induced oxidative damage. These results suggest that the aqueous extract of *D. oliveri* root bark may possibly be used as a promising natural product for the prevention of memory disorders. The result of this study supports the traditional claim of the plant's use for schizophrenia, epilepsy, and studies currently being conducted to delineate the toxicological profile of this plant.

## Data Availability

The data used to support the findings of this study are available from the corresponding author upon reasonable request.
